# Foreign Summary

**Published:** 1839-09-21

**Authors:** 


### FOREIGN SUMMARY.

*Process of Photography, or Sun-Painting.*—The history and details of M. Daguerre's photographic process, were communicated by M. Arago to the Academy of Sciences of Paris, at the sitting of the 19th of August. M. Daguerre's recipe is as follows:

"A copper-plate plated with silver, its surface well cleansed with dilute nitric acid, is exposed to the action of the vapour of iodine: this forms the first coating or ground, which is inconceivably thin, and requires to be perfectly even. The plate, thus prepared, is placed on the table of the camera obscura; and after remaining eight or ten minutes, (according as the subject or the degree of light may require,) is withdrawn: at this stage of the process, however, the most practised eye will not discern the slightest trace of the action of the light on the prepared surface. The plate is then exposed, in a proper apparatus, to the vapour of mercury; and, when heated to sixty degrees, the picture appears as if by magic. A singular, and hitherto inexplicable circumstance, requires to be noticed in this part of the process,—namely, that the plate must be in an inclined position; and that if it be placed directly opposite the aperture whence the vapour of the mercury escapes, the result will not be satisfactory. Lastly, the plate must be dipped in hypo-sulphate of soda, and afterwards well sluiced with distilled water: the operation is then complete.

"The cost of the plate must necessarily be considerable, and the chemical process requires nicety and skill; so that the expense of the photographic pictures will not be so trifling as might be supposed, especially when accidental failures are taken into account. By this process, it is to be borne in mind, the picture appears on the plate as it does on the camera,—that is, with its forms and shadows painted dark on a white ground. In the simpler process invented by Mr. Fox Talbot, by which the solar rays act on the prepared paper, called *photogenic*, the light and shades of the real objects are reversed, and the picture is painted white on a dark ground. Mr. Talbot's method of preparing photogenic or sensitive paper, consists in washing fine writing paper over first with a solution of nitrate of silver, then with bromide of potassium, and afterwards with nitrate of silver again; drying it at the fire after each operation. He also imitates etching on copper-plate, by smearing over a piece of glass with a solution of resin in turpentine, and blackening it by the smoke of a candle: on this ground the design is traced with the point of an etching needle, and the sensitive paper being placed behind the glass exposed to the sun, the rays of light, passing through the transparent lines, act upon the paper, and leave the design imprinted in a brown hue. The experiment can be repeated as often as may be desired. This last mentioned process, however, is but printing by sunlight from etching on glass: it is curious enough, but nothing compared to the drawings of light, where nature delineates her own image, reversed on paper; and this, again, is far inferior to the beautiful perfection of M. Daguerre's process, by which the external picture is depicted in miniature, light for light, and shade for shade, to the minutest gradation of each—only colourless."

*Election at University College and Hospital.*—Dr. C. J. B. Williams, (known in this country from his works on the chest,) has been elected Professor of the Principles and Practice of Medicine at University College, and Physician to the Hospital, vacant by the resignation of Dr. Elliotson. Among the candidates were Drs. Marshall Hall, Copland, and Craigie.

---

*Velpeau's Clinical Lectures on Ophthalmia. No. 1.*—GENTLEMEN,—Before I enter at length into the examination of the subject of which I am about to treat, I wish to make a few remarks respecting the history of this branch of surgical science, principally with the view of dispelling some erroneous opinions which seem to prevail, not only among foreigners, but even among many of our own countrymen: I allude to the reproach often made to France, that she has contributed but little to the advancement of Ophthalmology—that she has allowed other nations to take the lead, and has shown indifference to a class of diseases which has been cultivated with the greatest interest in neighbouring countries.

A cursory survey, however, of the history of ophthalmology, will show clearly that there is no real foundation for such an opinion, and that, although a much smaller number of special treatises on this subject has been published in France, during the last few years, than in England or in Germany, we are not at all behind these countries in our knowledge of this division of pathology. We must never forget that in medical science, as, indeed, in all others, it is not the number of authors, but the accuracy of their ideas, that ought to guide us in forming an opinion.

In all ages, diseases of the eye have attracted great attention; nor, indeed, can we be surprised at this, when we take into consideration the extreme importance of its functions, and the numerous maladies to which it is subject. No other organ of the animal economy, indeed, offers to our observation so many forms of disease; and this is at once accounted for when we consider the extremely complicated nature of its texture. In the eye we find nearly all the elements of our organization. In it we meet with cellular, muscular, vascular, fibrous, and nervous tissues. In it we find an apparatus for secretion and excretion; also tissues not to be observed in any other part of the body, such as the choroid membrane, the retina, the crystalline lens, and the vitreous humour. Each of these tissues must necessarily be subject to peculiar affections, manifesting themselves by peculiar symptoms, as is the case in all other regions of the body.

In Galen's time, a great number of diseases of the eye were already known. He says that many persons specially devoted themselves to this branch of the healing art; nor need we think this extraordinary, for then nearly every branch of medicine had become a prey to specialists. There were oculists, aurists, lithotomists, physicians for the chest, physicians for the abdomen, &c. &c.; and this is the state of things into which some would now wish us to return.

From Galen up to the sixteenth century, we find no important work on diseases of the eye. At that epoch France gave the signal—France opened the path of modern improvement. Guillemeau is the first who classed the different affections of the eye; this he did in an interesting treatise, in which he describes as many as one hundred and thirteen diseases, designating them by names mostly taken from the Greek authors.

It is not, however, until the end of the seventeenth, and during the eighteenth century, that we see those works appear which constitute an era in science. It was then that the writings of St. Ives, Maître Jaen, David, Brisseau, Guerin, Janin, Pellier, Wenzel, Demours, appeared in France; that of Scarpa, in Italy; those of Richter, Beer, Schmidt, Himly, in Germany; those of Woolhouse, Wathen, Ware, and others, in England. Hitherto France has, you see, her representatives among ophthalmologists, and their labours will bear comparison, in every respect, with those of foreigners. Even those who speak the most lightly of French surgery, yield to France the superiority during the eighteenth century. But where, they ask, are the men of our times whom you can compare with Jünghen, Ammon Ræsas, Jæger, in Germany; with Saunders, Wardrop, Travers, Mackenzie, in England; with Fabini, Quadri, Flarer, in Italy?

We cannot, I confess, bring forward so many pure ophthalmologists; but I appeal to your reason, is it necessary to be decorated with the name of oculist to have exact notions respecting diseases of the eye? Do not these affections belong to pathology in general? and ought not every good surgeon to be acquainted with this branch of his profession, as well as with all others? Absolute specialities ought not, in our days, to meet with the favour they met with formerly. Indeed, public opinion seems to be gradually becoming more enlightened in this respect; and we may still hope to see the time when specialities will fall entirely into the domain of charlatanry. You are aware, I presume, that I only allude to specialities as practised by those whose knowledge is confined to the branch of pathology they have embraced. I am far from wishing to cast any blame on the preference a practitioner may give to some branch or other of his profession. It would, I think, be difficult to find a man of science who has not some favourite subject to which he more particularly directs his attention.

If we look over the French periodicals, and the different works on surgery that have appeared in our own times, we shall certainly find many articles which the most celebrated ophthalmologists would not disown. Had we not a few years ago Demours junior, and Forlenze? Have we not now MM. Wenzel junior, Guillet, Faure, Gondret, Bourgot, Andrieux, Stæber? MM. Carron du Villard, Furnari, Rognetta, although of Italian extraction, profess among us French doctrines. Again, are Boyer and Dupuytren,—are MM. Roux, J. Cloquet, Sanson, Laugier, Marjolin, inferior to those surgeons of Great Britain or Germany? May I be allowed to say, that I myself have for many years paid great attention to diseases of the eye? In 1820 and in 1825, I published several articles on this subject. In 1831 I gave a series of special lectures on ophthalmology at La Pitié, some of which appeared in the periodicals of the day.

This I say merely with the view of proving that France has done as much as any other nation towards the advancement of this branch of pathology. Were I to say more, I should be obliged to enter into details which would carry me too far. I will, therefore, only add, that providing justice be rendered to France, I am perfectly willing to be generous to other nations.

In the course of these lectures, my opinions will often be at variance with the doctrines professed and adopted in other countries; I think it necessary, therefore, to announce, as briefly as possible, the doctrines to which I allude.

I shall occasionally find myself opposed to men of science, whom I esteem, some of whom I number among my friends. This consideration, however, cannot deter me from making my opinions public; every other feeling must give way when we are prompted by the interests of science and the welfare of humanity.

The school which reigns at the present time is that of Beer. Even M. Stæber has not escaped its influence. The doctrines professed by the pupils of this oculist repose on two principles, which must detain us for a few moments.

The first of these principles—the one which forms, as it were, the basis of the system—is, that diseases of the eye ought to be classed according to the natural system; that is, according to their physical symptoms. This mode of classification, which is the one adopted by botanists, appeared novel in its application to medical science; but were you to peruse the nosology of Sauvages, you would at once see that it is not necessary to go to Vienna or Berlin to meet with such an application of the natural system. The same may be said of Pinel. Is not this also the system of all the solidists—of the Broussaian school itself? Beer, it is true, only applied this principle to diseases of the eye, but M. Jæger extended it to pathology in general. This is the system he opposes to that of the French physiologists and organicians.

The second principle is, that the classification should also be founded on the nature of the disease; it may be considered the consequence of the first.

Such are the principles which have given birth to scrofulous, rheumatic, and catarrhal ophthalmia. Those of you who have followed my visits for any length of time, must have perceived that these denominations are founded on erroneous views. I do not mean to assert that a scrofulous constitution, or a syphilitic taint, will not exert great influence over diseases of the eye. The eye is no more shielded from such influence than any other organ. But I must be allowed to differ entirely from those who maintain that a disease of the eye assumes peculiar symptoms because the patient's constitution is modified by a general affection; and from those who pretend to judge of the constitution of persons from the ophthalmia presenting this or that symptom. Attentive observation of disease renders me every day more and more convinced that such opinions are entirely without foundation. Examine the patients now in our wards; many will offer you all the symptoms which the German school considers pathognomic of a special affection of the eye; yet you will find but very few among them under the influence of the general affection on which so much stress is laid by Beer. Those whom these cases might not convince, have it in their power to perform an experiment which would perhaps carry with it more weight than what is seen here. Nothing, indeed, is more more easy than to give rise in the healthy eye, by artificial means, to an inflammation assuming the symptoms of the scrofulous, the rheumatic, or the catarrhal form of ophthalmia.

Anticipating here the ideas I intend to develope in the following lectures, I will, in a few words, tell you my opinions on this subject. I believe the division of ophthalmia into scrofulous, rheumatic, and catarrhal, which the school of Beer is now attempting to establish, is altogether erroneous. These general affections exercise the same influence over diseases of the eye that they exercise over all other diseases. The error of Beer, and that of his followers, is simply this: they judge of the constitution by the appearance of the eye, whilst you ought to judge of the eye by the constitution. They show us the image inverted, if I may use the figure; I wish to place it in its proper position.

Scrofulous or rheumatic ophthalmia, &c., as understood by one of the representatives of the German school now among us,* does not, then, in my opinion, properly speaking, exist. The doubts which some of you may feel on this subject, will, I think, have vanished before we have brought our examination of these affections to a close. I shall endeavour to overthrow the opinions of the German school, not only because they are false, but also because they only serve to render more intricate a subject which is already extremely difficult.

To conclude these observations, I will say, that after having meditated frequently and seriously on the subject—after having made an immense number of observations at the bed-side of the patient—I think I am warranted in asserting, that the study of diseases of the eye ought to be directed by the same laws as that of all other branches of pathology.

Diseases of the eye may be divided into three classes:—

1st. Diseases of the eyelids.
2d. Diseases of the inner canthus.
3d. Diseases of the eye itself.

* M. Sichel, a private professor of ophthalmology, of high scientific attainments. M. S. has lately published a work, in which he entirely adopts the views of the German pathologists.

The diseases of the inner canthus having been sufficiently investigated during the last few years, I shall not enter into any details respecting them, referring you to what I have already published on the subject in my work on Operative Surgery, and in the Dictionnaire de Médecine.

With regard to diseases of the globe of the eye, I shall lay aside every thing relating to degenerations of tissue, confining myself to what authors have designated under the general term of ophthalmia.

*Inflammatory Diseases of the Eyelids.*

The different inflammatory diseases of the eyelids are known under the general term of *Blepharitis.* Blepharitis presents several varieties, determined by the nature of the tissue primarily affected, and by the variable intensity of the inflammation. I must again remind you, that, for the present, I do not take into consideration the constitution of the patient, or the different specific causes of disease.

The eyelids, considered in a general point of view, present an external or cutaneous surface, an internal or mucous surface, and a free margin. Between these you find a tissue, the structure of which must be well known before yon can form an exact notion of the inflammatory affections of the palpebræ. The most important anatomical feature is the laxity of the cellular tissue, uniting the muscular and cartilaginous portions, &c.

The anatomical structure of the eyelids might induce us to recognise an external, a median, and an internal blepharitis, as also a blepharitis of the free margin. Pathologists have, however, very justly excluded the two first from the list of inflammatory affections constituting ophthalmia: nevertheless, as inflammation here assumes a peculiar physiognomy, which it does not present in any other part of the body, the prepuce and scrotum excepted, I feel bound to say a few words on the subject.

When the inflammation is limited to the external surface of the eyelids, it offers, as I have already stated, the same characters as when it attacks the prepuce or scrotum. It is merely an erysipelatous or erythematous affection, which is to be treated by the means usually resorted to in these diseases. In the simple form, without any complication, the affection is so slight, that it is only necessary to point it out to you.

When, however, the intermediate layers are inflamed, the malady becomes more serious. The connection between these tissues and the inner surface of the eyelids is so intimate, that generally the inflammation, passing from one to the other, gives rise to blepharitis, properly so called. Thus, as we have not only to fear the consequences of the inflammation in the parts primarily affected, but also its extension to the palpebral conjunctiva, we ought at once to adopt every measure likely to arrest its progress.

This kind of blepharitis may assume two forms; it may be circumscribed, or it may be diffuse. When diffuse, it constitutes what is called phlegmonous erysipelas. In both cases, the best plan to extinguish the disease, and to avoid the usual consequences of phlegmonous erysipelas, is to scarify the parts, regulating the number of scarifications by the extent of the inflammation. These affections do not, in reality, deserve to be comprised under the head of ophthalmia. I shall not, therefore, enter into any further details respecting them, but at once commence the examination of blepharitis, properly so called—that is, of the inflammatory affections of the internal surface, and of the free margin of the eyelids.

Blepharitis, strictly so called, presents four principal forms, which are determined by the consideration of the structure of the parts primarily affected. Thus, when the inflammation is limited to the conjunctiva, I call it mucous blepharitis; when it occupies the Meibomian glands, glandular blepharitis. This second form admits of a variety, diphtherical blepharitis. The third form is not yet well known; the inflammation appears to be concentrated in the mucous follicles. This latter circumstance has induced me to give it the name of granular blepharitis. Lastly, I have called ciliary blepharitis that form in which the inflammation is situated at the base of the cilia. To these four principal forms we may add purulent blepharitis. We have therefore to study—

Mucous blepharitis.
Glandular ditto.
Granular ditto.
Ciliary ditto.
Purulent ditto.

Although I thus distinguish several forms of this disease, you must not suppose that they are always separate—always perfectly distinct from one another. This is by no means the case; they are, on the contrary, nearly always combined. Thus you will generally find two or three, sometimes even the whole four, present in the same eye. Each form, nevertheless, offers symptoms and characters peculiar to itself, which ought not to be confounded. We shall also find, shortly, that these distinctions are of great importance with regard to the prognostic and treatment of blepharitis, considered in a general view.

*London Medical Gazette.*

*La Charité Hospital Practice—Clinique of* M. Velpeau.

HYDROCELE.—VARICOCELE.—OPERATIONS.

*June 7th.* A man, aged 24, was admitted with hydrocele of some years standing, on the right side, and varicocele on the left. Both are excellent specimens of the diseases. General health good.

*8th.* To-day M. Velpeau, after removing the serous fluid from the tunica vaginalis, injected a solution of tincture of iodine in a much smaller quantity, which, after squeezing the scrotum, so as to ensure its application to the whole of the internal surface of the sac, he permitted to remain there. No other application was made use of.

*9th.* The varicocele was operated upon by introducing a single pin under the veins of the chord.

11*th.* The operation of hydrocele seems to be doing well. Tunica vaginalis well distended with lymph. There is a slough above the pin on the other side.

18*th.* Pin removed. The slough the size of a shilling.

24*th.* Ulcer left by the separation of the eschar is still without reparation.

*July 2d.* Doing well.

12*th.* Discharged cured.

BLOW UPON THE THIGH.—EFFUSION OF BLOOD.—DEATH FROM NEGLECTING TO TIE A SMALL ARTERY.

*June 9th.* Tonniard, aged 24. About a month ago he received a heavy blow over the lower third of the right femur, from a saw, which fell upon his limb. There was no wound externally; swelling soon followed, and he has been obliged to give up his employment.

There is a circumscribed tumour, firm, but admitting of an indistinct sense of fluctuation, in the situation of the external vastus muscle, where it is inserted into the patella. It is evidently not connected with the knee-joint. To-day M. Velpeau introduced a lancet into it, and some thin, bloody fluid escaped, with a few small coagula. The puncture was allowed to heal by the first intention, and a poultice was applied.

11*th.* More tumefaction, and a clearer sense of fluctuation in the tumour. M. Velpeau made a free incision into the tumour, and brought out a quantity of thick and and firm coagula. He then poked about the interior of the cavity in a very rude manner with his finger. To apply an emollient cataplasm. It will be interesting to see the result of this case. If the knee-joint becomes affected, probably the poking about the cavity with the finger may be said to be the chief cause.

12*th.* There has been considerable bleeding from the incision made yesterday. On lifting up the poultice, a large coagulum of blood was present in it. To-day the finger was again forced into the cavity, to the great pain of the patient, who complained loudly. The bleeding has most probably been afforded by some small arterial vessel. It does not bleed now; the wound and the cavity to be filled with charpie. The cavity is filled with coagula and grumous blood, which finds a very scanty and difficult egress.

13*th.* The abscess is again filled with blood from the little arterial vessel, which still continues to bleed, though now it is not easy either to see it or to seize it. Pulse is quick, 120. He has more pain in the knee, which is swollen, than before; has passed a very uneasy night on account of the pain in the part affected. The finger was again poked into the abscess freely.

14*th.* The man is very low indeed, without pulse at the wrist, and his face is exsanguined. The dressings and bed appeared to have been soaked with blood; and it would appear that the small artery which was cut the first day, has still continued to throw out blood. M. Velpeau looked for the vessel, but could not distinguish it. He introduced his finger again into the cavity, and showed that it had burrowed to an immense extent underneath the integuments and fascia of the lower part of the thigh, and was in close relation with the knee-joint. He made two counter-openings, one upon each side of the knee.

This poor young man died about noon, just five days after the opening of the extravasation of blood.

*After-death Appearances.*

An immense cavity underneath the vasti and crureus muscles; the surfaces irregular, and partially covered with fibrinous clots, which had become hard and dense, at the same time losing the colour of blood. It was said that this cavity had communicated with the knee-joint, but this is exceedingly doubtful, for there was no blood found in the articulation, nor any appearance of such having been the case; for during the life of the individual, and within a few hours of his death, M. Velpeau carefully examined the interior of the abscess with his finger, and did not find his way into the articulation. To-day, however, two fingers can be introduced through the supposed connection. There was no inflammation or other affection of the joint, which appeared healthy. The artery which had furnished the bleeding could not be traced. This poor young man died from severe hæmorrhage, consequent upon the neglect of tying a small integumental artery.—*Lancet.*

---

*Empyema, and remarkable Fistula of the Chest.* Reported by Dr. Rederle, Valée de Munster.—Frederick Wetzel, aged 19 years, a miner in the Black Forest, Germany, since his tenth year had always been well, excepting that he had frequent epistaxis.

On the 25th of April, 1834, he was attacked by pleuropneumonia, for which local and general bleedings were employed, blisters, nitre, calomel, and, finally, acetate of ammonia and tartarised antimony. The symptoms diminished, but after some days the breathing became more difficult, the pains returned in the left side of the chest from time to time, the cough recurred, the expectoration was purulent, his strength fell, he had hectic fever, night sweats, frequent shiverings, a dull weight on the left side, deep respiration excited cough, and the decubitus on the right side produced access of suffocation.

Infusion of senega root, digitalis, quinine, decoction of Iceland moss, yellow sulphuret of antimony, Dover's powder, and Seidlitz water, did not ameliorate the condition of the patient. In about eight days more, the right cavity of the chest was considerably prominent, œgophony was manifested, the respiratory bruit was null, and percussion elicited a dull sound. The cellular tissue of that side of the chest became œdematous, as also did the left foot.

On the 21st of June, between the fifth and the seventh ribs, there appeared a tumour of the size of a man's fist, adherent, and completely fluctuating. An opening made in it with a lancet produced two pints and a half of a thick and fetid pus. The patient, previously threatened with suffocation, was immediately relieved. At each dressing, which was renewed daily, there issued about a pound of pus. The quantity diminished, little by little, as the pus could not flow freely, while it became yet more fetid.

Some weeks after this a fresh fluctuation appeared, between the seventh and eighth ribs, and a new opening was made, sufficiently large to liberate the pus freely. A sound introduced into the cavity formed by a pseudo-membrane, *passed inwards and from before backwards*, to a depth of six inches. The sac was abundantly capacious. Care was taken to sustain the patient by tonics, and to evacuate the cavity always by means of lukewarm injections of chamomile tea. In 1835 and 1836, it was sought in vain to heal the opening by tincture of myrrh, decoction of quinine, salicine, oak-bark, and madder-root. During the three years that the fistula remained open, it was impossible to prevent the entrance of air, which, however, had no other inconvenient effect than that of rendering the respiration more difficult. The pus which issued at this time seldom exceeded two table-spoonfuls in quantity. Very serious hæmorrhages within the purulent cavity threatened life on many occasions in August and December, 1836, and June, September, and December, 1837. These appeared to have supplanted the epistaxis to which the patient had been subject. The quantity of blackish fluid and coagulated blood sometimes reached a pint and a half. On stopping the mouth of the fistula, the blood occasioned severe oppression, and even cough, with sanguineous expectoration. As soon as the issue was re-established, these symptoms ceased.

From time to time the patient is troubled with abdominal symptoms, which a purgative removes. He has often, also, palpitations of the heart; the urine is then diminished, and the hands and feet are œdematous. Digitalis always disperses these symptoms. At present, as during the preceding summer, the patient feels very well, attends to his domestic duties, and even walks nearly three-quarters of a mile at a time.—*Ib.*, *from Medicinische Annalen*, part 4, vol. iv.

---

*Experimental Investigation into the Functions of the Eighth Pair of Nerves.* By John Reid, M. D.—This series of experiments was performed in continuation of those formerly published by the author, of which we inserted an abstract in our First Volume, (Nos. 8 and 9, pp. 128, 140.)

The experiments related in this paper almost entirely concern the functions of the pulmonary and gastric branches of the par vagum—those *quæstiones vexatæ* of neurology. A few additions are made, however, on the subjects of the former series; to these we shall first advert.

*Glosso-pharyngeal Nerve.* Dr. Reid has now frequently succeeded in producing *the usual muscular movements* of deglutition, by pinching the trunk of this nerve. He had before only produced convulsive twitchings. As he has fully proved that it is not a motor nerve, but acts on the muscles by reflexion, it may evidently be regarded as the principal excitor of the movements of deglutition. That irritation of the trunk of the nerve should have less power of exciting them than impressions made on its extremities, harmonizes well with what we learn from other sources of the character of this class of actions.

*Pneumogastric Nerve.* With regard to the general character of this nerve, Dr. R.'s subsequent experiments have fully confirmed his former opinion, that it ministers to sensation; indications of severe suffering being given when its trunk is pinched, cut, or stretched. He finds an explanation of the negative results which some experimenters have obtained, in the fact that some animals appear much less sensitive to pain than others, even of the same species. "I have experimented on dogs which have endured the incisions necessary to expose the sheath of the carotid artery, without any apparent uneasiness." Magendie has made similar observations: "Such facts ought to make us hesitate before arriving at negative conclusions upon the sensibility of nerves, and forcibly point out a serious error to which limited observations are liable."

*Laryngeal branches.* On the functions of these, also, Dr. R. has obtained additional proof of the correctness of his views. He has found that, after the first paroxysm occasioned by division of these nerves, a period of quiescence and freedom from dyspnœa often supervenes, the respirations being performed with ease as long as the animal remains at rest; but an unusual inspiratory movement, such as takes place at the commencement of a struggle, induces immediate symptoms of suffocation, the current of air carrying inwards the arytenoid cartilages, rendered passive by the paralysed state of their muscles, so as to obstruct the opening of the glottis.

*Cardiac branches.* From seven experiments upon dogs, the following inference is deduced, which is opposed to that of M. Brachet: "After section of the vagi, the pulsations of the heart may not only be quickened by muscular exertion, but also by mental emotions. Though in all probability the vagi are the usual channels through which mental emotions affect the heart, yet it appears that this may also take place through the medium of the ganglionic system of nerves."

*Pulmonary branches.* The author confirms his former statement that lesion of one of the pneumogastrics does not necessarily or even generally induce disease of the lung on that side; and he then discusses at much length the effects of lesion of this nerve upon the *respiratory movements*. A large number of experiments were performed to determine this point; and it was an object in these to avoid the disturbance arising from the paralysis of the laryngeal muscles as much as possible. The details of Dr. R.'s method of operating are given by him, that those who wish to test the value of the facts ascertained by him may repeat his experiments in a similar manner. "It is not so much," he remarks, "the frequency of false observations that we have to complain of in medical science, as the errors which arise from making these observations under dissimilar circumstances, and from drawing general conclusions from insufficient data."

The first and most positive effect of division of the pneumogastric nerves upon the respiratory movements, is a decided *diminution in their frequency.* The number of inspirations soon after the operation was, in the majority of the experiments, reduced about one-half. In some cases the number of respirations was afterwards still further reduced, while in others it varied at different periods; and in a few it became more frequent shortly before death. The movements themselves are performed more slowly, in a prolonged and heaving manner, so as to give rise to the idea entertained by some experimenters, and by Dr. R. himself in the first instance, that they are more numerous. It is sufficiently evident, from these experiments, that the vagi are important nerves in transmitting those impressions to the medulla oblongata which excite the respiratory muscular movements. Dr. R. then goes on to prove, by experiments, that Dr. Cruveilhier and Dr. M. Hall are incorrect in asserting that respiration will not go on if the cerebrum and cerebellum are destroyed and the pneumogastrics divided; a contrary result having been obtained by him. The removal of the encephalon diminishes the frequency of these movements, however, whether before or after the section of the vagi. In kittens of a day old they were at first about one hundred per minute; after the destruction of the brain and cerebellum they fell to forty; and on cutting the pneumogastrics they instantly fell to between three and four in the minute, and continued so for some time. It is thus proved that, although the pneumogastric nerves are the *principal,* they are not the *sole* excitors of the respiratory movements; and that there are others which act through a channel in which the encephalon is not concerned. In one of the experiments the spinal cord was divided high up in the neck after the removal of the brain and the division of the pneumogastrics. Respiratory movements were still performed, though with diminished frequency. This fact seems to confirm the doctrine of Dr. M. Hall, relative to the function of the cutaneous nerves, especially those of the face, as excitors of respiration. It is not certain, however, how much of the effect is to be attributed to the sympathetic, which, as has been recently proved by microscopic examination, contains many filaments of the cerebro-spinal system. The diminution in the frequency of the respiratory movements subsequent to division of the vagi has been noticed by other observers; but its constancy has not previously been shown, nor has it been pointed out that it *instantly* supervenes, (unless the animal be excited by struggling,) and thus cannot result from the circulation of dark blood through the nervous centres.

The next question discussed is that of the *motor* character of the pulmonary branches of the vagus. Regarding this Dr. Reid has not been able to obtain any positive evidence on either side. Certain pathological phenomena would lead to the conclusion that these nerves are capable of exciting muscular contraction in the bronchial tubes; but experiment does not bear out this supposition, though it does not forbid it. The affirmative results obtained by M. Brachet, are not regarded by Dr. R. as worthy of confidence. With regard to the *sensory* functions of these branches, Dr. R. is of opinion that, although they are concerned in producing the sensation of "le besoin de respirer," this is *not* annihilated after their section, since animals will even then exhibit great uneasiness if the access of air to their lungs be prevented. The fallacy of the contrary results obtained by Brachet and Mr. Grainger, is pointed out. We would suggest that this uneasiness may be produced by impressions transmitted from other parts of the system as well as the lungs, when imperfectly arterialized blood is circulating through the systemic capillaries, just as the sense of hunger seems to depend not only upon the emptiness of the stomach, but upon the demand for nutrition existing in the system at large. The sensibility of the mucous membrane of the trachea and bronchi appears to be impaired by section of the vagi; but it is always much inferior to that of the larynx; and the experiments made to test it are liable to a remarkable fallacy, arising from the fact that the air-passages are easily brought to tolerate stimulants which at first excite great uneasiness.

*Morbid changes in the Lungs* consequent upon section of the pneumogastrics. The real character of these, and the mode in which they are induced, constitute a very interesting subject of investigation; and one whose importance in pathology is at once apparent. We are inclined to place the more reliance upon Dr. Reid's statements, as his office as Pathological Clerk at the Royal Infirmary of Edinburgh gives him peculiar opportunities of becoming conversant with morbid appearances, and induces accuracy in the statement of them. In fifteen out of seventeen animals experimented on, the lungs were found more or less unfit for the healthy performance of their functions. Of the two animals in which the lungs were found healthy, one died after the completion of the eighth day, apparently from inanition; the other was killed after the twelfth day, when it was apparently in perfect health. "The most common morbid changes found in the lungs of these animals was a congested state of the blood-vessels, and the effusion of frothy serum into the air-cells and bronchial tubes. In eight out of the fifteen these changes were strongly marked. In some portions of the lungs the quantity of blood was so great as to render them dense. The degree of congestion varied in different parts of the same lung, but was generally greatest at the most depending portions. The condensation was generally greater than what could be accounted for by the mere congestion of blood in the vessels, and probably arose from the escape of the solid parts of the blood into the tissue of the lung." In some instances the condensation was so great that a considerable portion of the lungs sunk in water and did not crepitate; but they did not present the granulated appearance of the second stage of ordinary pneumonia. In five cases in which the animals had survived a considerable time, portions of the lungs exhibited the second and even the third stages of pneumonia, with puriform effusion in the smaller bronchial tubes; and in two gangrene had supervened. "One of the most important points to ascertain in an investigation of this kind is the first departure from the healthy state; to decide whether the effusion of frothy, reddish serum, by interfering with the usual changes of blood in the lungs, *causes* the congested state of the pulmonary blood-vessels and the laboured respiration; or whether this effusion is the *effect* of a previously congested state of the blood-vessels." In several of the experiments but a very small quantity of frothy serum was found in the air-tubes, even when the lungs were found loaded with blood, and when the respiration before death was very laboured. "This naturally leads us to doubt whether the frothy serum is the cause of the laboured respiration and the congested state of the pulmonary vessels in those cases where it is present, though there can be no doubt that, when once it is effused, it must powerfully tend to increase the difficulty of the respiration, and the impeded circulation through the lungs." Dr. R. has satisfied himself of an important point which has been overlooked by others, "that this frothy fluid is not mucus, though it is occasionally mixed with it, but is the frothy serum so frequently found in cases where the circulation through the lungs has been impeded for some time before death." From this and other facts Dr. R. concludes "that the congestion of the blood-vessels is the first departure from the healthy state of the lung, and that the effusion of frothy serum is a subsequent effect." What, then, is the cause of this congestion, and of the retardation of the movement of the blood? We fully agree with Dr. R. in considering the diminished frequency of the respiratory movements, and the consequent check to the aërating changes, as sufficient to account for it, since "it is an established fact that the flow of the blood through the lungs is dependent upon the continuance of the respiratory process." It has been supposed by some to be due to paralysis of the muscular fibres of the bronchial tubes; after every endeavour to obtain distinct evidence of this, however, he has been unsuccessful, though he does not deny the possibility of such an influence. Several cases of disease in man are mentioned by him, in which the lungs presented after death a condition similar to that observed in the lower animals after section of the vagi; and in these the respiratory movements had been much less frequent than natural during the latter part of life, owing to a torpid condition of the nervous centres. He controverts the opinion of Wilson Philip, that the effusion is the result of a morbid secretion immediately consequent upon interruption of nervous influence, and points out how galvanism may operate in preventing it. The true mucus of the bronchial membrane has always been found, except when inflammation was present. The congestion of the vessels sufficiently accounts not only for the effusion of serum, but also for the tendency to pass into the inflammatory condition presented by the lungs as by other organs similarly affected. We are disposed to think, however, that this is not the whole truth, but that the interruption of innervation here, as in other cases, predisposes to inflammation, although it does not actually produce it. "The experimental history of the par vagum," remarks Dr. Reid, "furnishes an excellent illustration of the numerous difficulties with which the physiologist has to contend, from the impossibility of insulating any individual organ from its mutual actions and reactions, when he wishes to examine the order and dependence of its phenomena." Nothing, indeed, can be more various than the statements made by different experimenters of the results of their inquiries, of which an instructive summary is given by Dr. R. These differences, no doubt, arise from varieties in the conditions of the experiment; and the number of cases related in this memoir has enabled the author to point out several of these varieties, which had been unsuspected by his predecessors, whose experience was more limited. These are investigations in which no useful inference can be drawn from one or two experiments only; to avoid all sources of fallacy a large number must be made, and the points in which all agree separated from others on which there is a variation of results; and it must then be inquired to what the latter is due.

These observations apply equally to the other question which has been made by Dr. Reid a special subject of investigation—the function of the *gastric branches* of the vagus nerve—on which there has been a difference of opinion no less remarkable than on that just discussed. The experiments of Dr. Reid do not furnish grounds for positive conclusions on the subject; but they furnish important corrections of the results presented by others. He has frequently succeeded in exciting muscular contractions in the stomach by irritation of the nerve; but he has also frequently failed. It may be questioned, however, whether these movements are produced by the direct influence of the nerves, or whether they are excited by the contractions of the œsophageal filaments, which are unquestionably stimulated through the nerves. When these movements of the stomach are thus artificially induced, they commence at the cardiac orifice, and propagate themselves over a greater or less extent of the left portion of the viscus; they are evidently slower, more prolonged, and more vermicular than those of the œsophagus. These movements of the stomach, however, may take place after the section of the vagi, as is proved by some experiments presently to be mentioned, in which the food had been digested and propelled into the intestines, subsequently to this operation. "That lesion of the vagi is generally followed by vomiting, (in those animals susceptible of it,) loathing of food, and arrestment of the digestive process, has been incontrovertibly proved by numerous experimenters. That perfect digestion may occasionally take place after division of the vagi in the neck, even when the cut ends of the nerves are kept apart from each other, we are also fully convinced. In four of the seventeen animals experimented on for the purpose of examining the morbid changes in the lungs, we obtained sufficient evidence of the restoration of the digestive process." This evidence consisted in the acquisition of flesh and strength by the animal, which had at first suffered much from the effects of the operation; the vomiting of half-digested food, permanently reddening litmus paper; the disappearance of a considerable quantity of alimentary matter from the intestinal canal, and the existence of chyle in the lacteals. In all these cases it is to be observed that the digestive powers did not appear re-established until the fifth day after the operation; having been in these animals, as in those which died before that period, completely arrested in the first instance. This fact accounts for the contrary results obtained by other experimenters; as it is only when circumstances favour prolonged life that such restoration can be manifested. Dr. Reid states that seven of the seventeen experiments were performed before he obtained any evidence of digestion; and that the four which furnished this followed one another almost in succession, so that it is easy to explain how some experimenters have been more successful than others when the number of their experiments is limited.

Dr. Reid next considers the effects of lesion of the vagi upon the *sensations of hunger and satiety*, and shows that these are not abolished by section of these nerves, as Brachet and others have maintained, though very probably impaired. From this and other facts he is led to the conclusion that these sensations, though referred to the stomach as their seat, originate in impressions made upon the nerves of the system at large, and have reference rather to its requirements than to the fulness or emptiness of the digestive cavities. He then adverts to the fact that the *secretion of gastric juice*, of the true acid character and solvent powers, is not always checked by section of the nerves; and he quotes the statement of Müller, that where it does appear to cease, the application of galvanism has not succeeded in re-establishing it. We hope, therefore, that physiologists will, ere long, be tired of quoting the oft-cited experiments of Dr. W. Philip in proof of the *dependence* of this secretion upon nervous influence, and the identity of the latter with galvanic electricity. The usual mucous secretions, too, were always found; and the inflamed appearance of the mucous membrane of the stomach, described by Gendrin, was never noticed. Another series of experiments was performed by Dr. Reid, with the view of testing the validity of the results obtained by Sir B. Brodie, relative to the effects of section of the nerves upon the secretions of the stomach, after the introduction of arsenious acid into the system. With this view he made five sets of comparative experiments, employing in each two dogs as nearly as possible of equal size and strength, introducing the same quantity of the poison into the system of each in the same manner, but cutting the vagi in one, and leaving them entire in the other. This *comparative* mode of experimenting is obviously the only one admissible in such an investigation. Its result was in every instance opposed to the statements of Sir B. Brodie; the quantity of the mucous and watery secretions of the stomach being nearly the same in each pair of animals subjected to experiment; so that they can no longer be referred to the influence of the eighth pair of nerves. Moreover, the appearances of inflammation were, in four out of the five cases, greatest in the animals whose vagi were left entire; and this seemed to be referrible to the longer duration of their lives after the arsenic had been introduced. The results of Sir B. Brodie's experiments may, perhaps, be explained by the speedy occurrence of death in the subjects of them, consequent, it may be, upon the want of sufficiently free respiration.

The following is Dr. Reid's general conclusion from his experiments on the influence of the eighth pair upon the secretive processes of the stomach, which, we think, gives a peculiarly judicious view of the nature of nervous influence upon these processes in general. "These experiments ought to be viewed in two different aspects; for while they show that the integrity of those nerves is not a condition absolutely necessary for the performance of secretion in the stomach, they yet prove that the secretions usually poured into the interior of that organ may be modified, in a most important manner, by causes acting through these nerves. We have here two perfectly separate and distinct propositions, which have sometimes not been clearly distinguished from one another. The difference between them may be illustrated in a very simple manner. The movements of a horse are independent of the rider on his back—or, in other words, the rider does not furnish the conditions necessary for the movements of the horse—but every one knows how much these movements may be influenced by the hand and heel of the rider." "When we extend our investigation into the manner in which the function of secretion is performed to the whole range of the vegetable and animal kingdoms, we can, I think, have little difficulty in arriving at the conclusion that the nerves exercise over secretion not an uniform and essential, but an occasional and controlling influence."

The *cause of death* after section of the vagi may usually, therefore, be looked for in the changes which take place in the lungs; occasionally, however, it results from inanition, produced by the derangement of the digestive organs. It is of course understood that means have been taken to insure the access of a sufficient quantity of air into the lungs.

The following is a summary of the results obtained by Dr. Reid, as to the functions of the different portions of the pneumogastric nerve.

The *pharyngeal branches* are entirely or almost entirely motor, and move the muscles of the pharynx and soft palate.

Of the *laryngeal* branches, the superior are almost entirely sensory, supplying the mucous surface of the larynx and part of the pharynx. The few motor filaments they contain are distributed to the cricothyroid muscles. The inferior laryngeals are principally motor, and regulate the movements of the muscles attached to the arytenoid cartilages; they also furnish sensory filaments to the trachea, and a few to the larynx and pharynx. When an irritation applied to the mucous surface of the larynx produces contraction of the muscles which approximate to the arytenoid cartilages, it is by a reflex action through the spinal cord, in which the superior laryngeals probably act as the afferent nerves, and the inferior as the efferent or motor.

The *œsophageal* filaments are partly afferent and partly motor, the contact of food producing muscular contraction by reflexed stimulation. The degree in which this mode of action extends down the œsophagus seems different, however, in different species of animals.

The *cardiac* branches do not seem necessary to the sympathy between the heart's actions and mental emotions, although probably the usual channel of it.

The *pulmonary* branches seem to be the nerves chiefly concerned in transmitting to the medulla oblongata the impressions which excite respiratory movements, and are thus principally afferent nerves. It is possible that they contain motor filaments also.

The *gastric* branches appear to influence both the muscular movements of the stomach and secretions poured from its inner surface; but neither of these changes are absolutely dependent upon their constant influence.—*Brit. and For. Med. Rev., condensed from Edinburgh Med. and Surg. Journal.* April, 1839.

---

*On Chlorosis accompanied by Menorrhagia.* By M. Trousseau.—The coincidence of diminished or suppressed menses with chlorosis is so common, that the fact is frequently overlooked that a chlorotic state may exist in conjunction with menorrhagia; in both we have extreme paleness, discoloration of the blood, a tendency to dilatation of the heart, *bruit de soufflet* in the principal arteries, and neuralgia in different regions; when the menstrual flow is deficient, these symptoms are said to arise from chlorosis; when it is superabundant, from anemia. We frequently find that chlorosis may be traced in the first instance to a copious nose-bleeding, or to any other unusual loss of blood, arising accidentally or produced therapeutically; so that a transitory anemia will give rise to permanent discoloration and increased liquidity of the blood. This altered state of the circulating fluid is itself sufficient to give rise to hæmorrhage, which, thus occurring alternately as cause and effect, does not allow the patient to escape from its evil consequences. This form of chlorosis is, however, much less frequent than than that which is attended by amenorrhœa; it forms only one-fourth of the cases occurring in adults, and perhaps one-twelfth in those of young girls. In twelve cases collected by M. Trousseau, there was no serious lesion of the uterus. It would appear at first view that the treatment adopted for amenorrhœal chlorosis would not be suitable for the menorrhagic form; thus, iron, which is given with so much success to restore the menstrual secretion, would hardly appear appropriate when that secretion is in excess: but is not the effect of iron on chlorotic patients rather tonic than emmenagogue? We generally find the health partially re-established before the menses return: the complexion regains its natural tint, the depraved appetite, the pain at stomach, the palpitation of the heart cease, and the *bruit de soufflet* in the arteries is lost, so that the patient frequently recovers the appearance of health before the menses reappear. Presently this secretion is re-established, in consequence of the general return of the system to a healthy state. Iron, then, is only emmenagogue because it is tonic or reconstituent. Re-established health is not owing to the returning menses, but the contrary. On this view we shall have no difficulty in conceiving that preparations of iron will be of the greatest utility in menorrhagic chlorosis, in which it will operate both as tonic and hemostatic. Giving freely, the preparations of iron between two menstrual periods, we shall find the blood rapidly regain its lost constituents of colouring matter and fibrine, and the secretion will be much less abundant, but more coloured. If more powerful anti-hæmorrhagic means are required, the ergot of rye will be very useful. Uterine hæmorrhages are generally more violent in the night than during the day, and they occur with the greatest violence about 4 or 5 o'clock, A. M. Without pretending to account for this singularity, we shall find it highly useful to give a dose of *recently-powdered* ergot (gr. xv. to ℈j.) in the evening, and again about 4 in the morning. In many cases, the more simple and agreeable administration of acids will be sufficient; of these, the best is the citric, given in its natural form of lemon-juice. If these means are necessary, the chalybeate must be suspended during the menstruation, and afterwards continued whilst any symptoms of chlorosis remain.—*Ib., from Journal des Connaissances Medico-Chirurgicales.*

---

*Congenital Absence of the Liver.*—This rare malformation was found, by Dr. Kieselbach, in a human embryo, which was, in all other respects, well formed. The umbilical vein passed through the umbilicus to the part which the liver usually occupies, without dividing; there the portal vein received it, and divided into two branches, one of which passed to the vena cava, but the other divided into innumerable ramifying branches, which terminated blindly. There were no traces of hepatic veins. One cannot, therefore, regard the flocculent division of the branches of the umbilical vein as residue of a liver which had once existed, and at a later period had, from some cause, wasted. The case is probably to be regarded as one of those rare arrests of development in which one of the most important organs is not formed, but in which a vascular growth occupies its place.—*London Medical Gazette, from Froriep's Neue Notizen, No.* 159.

---

*Report of the Results of Revaccination in the Prussian Army in the year* 1838, *drawn up from Official Documents.* By Dr. Lohmeyer.—Total number of individuals inoculated (revaccinated) 42,041, of which number there were cicatrizes from former vaccination—

| | |
|---|---|
| distinct, in . . . | 33,819 |
| indistinct, in . . . | 5,645 |
| none, in . . . . | 2,577 |

The resulting vaccination was

| | |
|---|---|
| regular, in . . . | 19,117 |
| irregular, in . . . | 8,672 |
| did not take, in . . . | 14,252 |

In the cases of failure the vaccination was repeated with effect, in . 2,306
without effect, in . 10,424

Of the individuals in whom the disease was perfectly regular, there were from

| | | |
|---|---|---|
| 1 to 5 | pustules in | 8,787 |
| 6 to 10 | " | 5,581 |
| 11 to 20 | " | 4,056 |
| 21 to 30 | " | 693 |

Of individuals revaccinated with effect in the present and former years, there were affected, during 1838,

| | |
|---|---|
| with varicella, . . | 19 |
| " varioloid, . . | 10 |
| " true small-pox, . | 2 |

It appears, from the preceding statement, that the results of revaccination in the army, during the year 1838, closely resemble those observed in 1837, in both years about 45 in the 100 exhibiting true vesicles running a regular course. The resemblance would have been still greater, but for the circumstance that many men were included who, according to their own account, had been previously revaccinated at their own homes, but who exhibited no marks on their arms; these were almost always among the failures. According to the reports of the medical officers, there were also many cases in which the development and course of the disease were disturbed, chiefly through carelessness on the part of the individuals in allowing the pustules to be broken or otherwise injured.

The opinion formerly pretty prevalent that, failing lymph immediately from the cow, the only proper matter for vaccination is that taken from children with good pocks, has now few supporters among the military surgeons, as they have ascertained by manifold experience that the lymph from well-formed pocks of adults, whether in the first or second vaccination, produces as fine and regularly-proceeding vesicles as that from children. Accordingly, it is the practice of most of the medical officers, only in the commencement of the revaccination to use lymph from children. Many even, like the Wurtemburg vaccinators, give the preference to the latter for communicating the disease to adults.

Inoculation with dry, preserved lymph, was in a great degree ineffectual. In a child vaccinated with lymph of this kind, there appearing no result, it was again vaccinated, eight days later, with fresh lymph from the arm, after which not only all the latter punctures, but also two of the former took, and exhibited good and regularly-proceeding pocks. In the case of a man of the 6th artillery brigade, the pocks did not appear until six weeks after the inoculation, (two on the right and three on the left arm,) and their development was accompanied by so much inflammation in the vicinity, and gave rise to so much fever, that it became necessary to remove the patient into the hospital.

In several individuals, natural small-pox appeared soon after the vaccination. In two cases this happened before the third day, and in these the vaccination had no result. In two other cases, on the contrary, the modified small-pox (varioloid) appeared when the vaccine vesicles were in perfection, and then they sustained no alteration in their progress.

The whole number of small-pox cases in the army, in 1838, were 111; 56 being characterized as varicella, 43 as varioloid, and 12 as true variola; in this number are included the 31 cases formerly mentioned as occurring after successful revaccination. Seven cases of the small-pox were fatal, but no fatal cases occurred among the 31 just mentioned; in all of whom, on the contrary, the disease was mild, and indeed quite insignificant. The greater number of cases of small-pox (as was also the case in former years) took place in recruits shortly after joining the army, and before revaccination could be employed. Some of the older soldiers, however, were also attacked with the disease in some of its forms; for instance, some of the subordinate officers who had been previously revaccinated without effect, or who had entered the army previously to the introduction of the practice of revaccination. Most of the cases of natural small-pox occurred in the 7th division, viz. 37, and for the most part in the garrison of Minden. In the 4th division not a man failed with small-pox, although the troops mixed more or less with the inhabitants of their stations or of the vicinity. This favourable result is principally to be attributed to this circumstance—that in the district of the 4th division the recruits joining the army in the autumn of 1837 were, for the most part, subjected to revaccination immediately on their arrival; whereas, in other cases, it has been customary to put off the revaccination of such recruits, at least in a considerable proportion, until the spring.—*Medicinische Zeitung*, May 8, 1839; *and Brit. and For. Med. Rev.*, No. 15.

